# Morphological, Genome and Gene Expression Changes in Newly Induced Autopolyploid *Chrysanthemum lavandulifolium* (Fisch. ex Trautv.) Makino

**DOI:** 10.3390/ijms17101690

**Published:** 2016-10-09

**Authors:** Ri Gao, Haibin Wang, Bin Dong, Xiaodong Yang, Sumei Chen, Jiafu Jiang, Zhaohe Zhang, Chen Liu, Nan Zhao, Fadi Chen

**Affiliations:** 1 College of Horticulture, Nanjing Agricultural University, Nanjing 210095, China; 2013204031@njau.edu.cn (R.G.); hb@njau.edu.cn (H.W.); 2013204035@njau.edu.cn (B.D.); 2014104120@njau.edu.cn (X.Y.); chensm@njau.edu.cn (S.C.); jiangjiafu@njau.edu.cn (J.J.); 201420402@njau.edu.cn (Z.Z.); 2014204030@njau.edu.cn (C.L.); 2015204028@njau.edu.cn (N.Z.); 2 Department of Horticulture, Agricultural College Yanbian University, Park Road 977, Yanji 133002, China

**Keywords:** *Chrysanthemum lavandulifolium*, morphology, SRAP (sequence-related amplified polymorphism), AFLP (amplified fragment length polymorphism), MSAP (methylation sensitive amplified polymorphism)

## Abstract

Autopolyploidy is widespread in higher plants and plays an important role in the process of evolution. The present study successfully induced autotetraploidys from *Chrysanthemum lavandulifolium* by colchicine. The plant morphology, genomic, transcriptomic, and epigenetic changes between tetraploid and diploid plants were investigated. Ligulate flower, tubular flower and leaves of tetraploid plants were greater than those of the diploid plants. Compared with diploid plants, the genome changed as a consequence of polyploidization in tetraploid plants, namely, 1.1% lost fragments and 1.6% novel fragments occurred. In addition, DNA methylation increased after genome doubling in tetraploid plants. Among 485 common transcript-derived fragments (TDFs), which existed in tetraploid and diploid progenitors, 62 fragments were detected as differentially expressed TDFs, 6.8% of TDFs exhibited up-regulated gene expression in the tetraploid plants and 6.0% exhibited down-regulation. The present study provides a reference for further studying the autopolyploidization role in the evolution of *C. lavandulifolium.* In conclusion, the autopolyploid *C. lavandulifolium* showed a global change in morphology, genome and gene expression compared with corresponding diploid.

## 1. Introduction

Polyploidy is a universal phenomenon in higher plants. Approximately 30%–70% of angiosperms are polyploids or cryptic polyploids [1], having undergone at least one event of whole genome duplication (WGD) in their evolutionary history. Polyploidy can be classified into autopolyploidy and allopolyploidy. The former results from doubling of a species’ chromosome complement, whereas the latter is the combination of two or more sets of different species [2]. Polyploidy has significant utility for adaptation to special conditions, such as drought and waterlogging, saline and alkaline stress, pest resistance, and others [3,4]. Polyploids can exhibit novel phenotypes that are not present in their diploid progenitors or exceeded the range of the contributing species in terms of certain traits, such as organ size and biomass, apomixis, and others [5,6]. All of these factors can give polyploids heightened chances to survive in new environments and can improve their agricultural output and quality. Polyploidization is a frequent process in plant evolution. However, we know very relatively little about the related genetic and epigenetic modifications, which could relieve the “genomic shock” caused by polyploidization [7]. There have been extensive studies regarding allopolyploidy, in which the genome structure is changed. Synthetic allopolyploids and parent suffer a measurable degree of sequence elimination, which broadly affects gene expression patterns [8,9,10,11]. Wang et al. [12,13] have already evidenced changes of genetic and epigenetic in wide crosses between species in Asteraceae and genomic and transcriptomic alterations in allopolyploids may accelerate the evolutionary process.

Autopolyploids consist of duplicate genes. Therefore, lacking necessary evolution potential compared with allopolyploids, autopolyploids were commonly considered an endpoint or “dead end” of an evolutionary path [14]. However, increasing evidence indicates that the actual appearance of autotetraploid plants in nature might be significantly underestimated [15,16]. The latest studies indicate that genome duplication confers features to adapt to new environments, e.g., hexaploid plants survive better than autotetraploid ones [17]. It appears that chromosome doubling to form polyploidy actually plays an important role in evolution.

Polyploidization can induce epigenetic changes in genomes. Epigenetic modifications, which include DNA methylation, histone modification, chromatin remodeling and micro ribonucleic acid (microRNA) activity, involve heritable changes in gene expression without changes in the nucleotide sequence [18,19]. Methylation levels exhibit different changes in diverse species [20,21,22]. Dar et al. [23] reported that the DNA methylation level in tetraploid *Phlox drummondii* was higher than in the diploid. Rodriguez et al. [24] indicated that diploid and tetraploid *Paspalum notatum* had no difference in the proportion of total methylation level but the methylation patterns were significantly more variable in tetraploids. Changes in DNA methylation are a common phenomenon in neopolyploids and has been associated with gene activation and silencing [25]. However, there is an opposing report. Church et al. [26] have demonstrated that the ploidy level is irrelevant to gene silencing or novel gene expression in autopolyploid lineages of *Helianthus decapetalus*. Much gene silencing and activation occur in allopolyploid plants but may not occur in the process of autopolyploidization [27,28]. Current studies are unable to provide irrefutable evidence, and additional studies are needed to investigate the changes in methylation associated with autopolyploidization.

Asteraceae is the largest family of flowering plants, consisting of approximately 25,000 species [29], with a large representation of polyploids. Although polyploidization is a fundamental biological process, it is relatively underexplored, and little is known about genome changes associated with WGD in Asteraceae. Here, we present results on how WGD alters genomic and transcriptomic changes by examining diploid *Chrysanthemum. lavandulifolium* and its autotetraploids through DNA-SRAP, DNA-MSAP and cDNA-AFLP fingerprinting.

## 2. Results

### 2.1. Chromosome Number and Morphology Analysis

**Figure 1:**
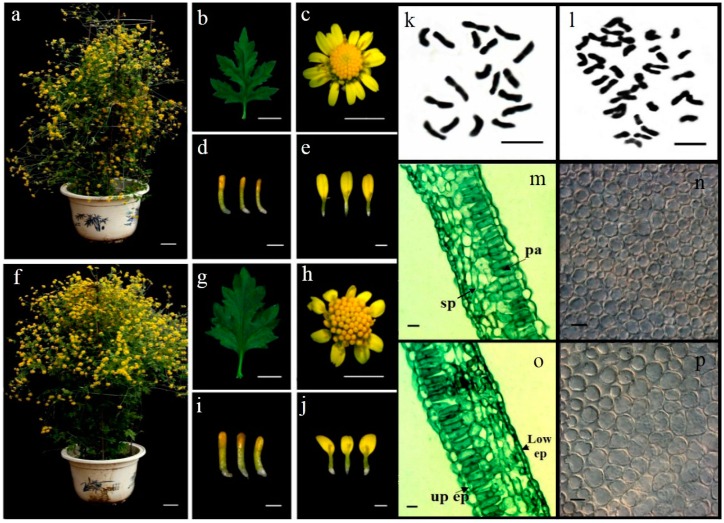
Morphology and chromosome number of diploid and tetraploid *Chrysanthemum lavandulifolium:* (**a**–**e**,**k**,**m**,**n**) diploid plant, leaf, inflorescence, tubular florets, ligulate florets, anatomical structure of leave, cell of palisade tissue first layer, and number of chromosomes of diploid plant; and (**f**–**j**,**l**,**o**,**p**) tetraploid plant, leaf, inflorescence, tubular florets, ligulate florets, anatomical structure of leave, cell of palisade tissue first layer, and number of chromosomes of tetraploid plant. up ep, upper epidermis, low ep, lower epidermis, sp, spongy tissue, pa, palisade tissue. (**a**,**f**) bars = 5 cm; (**b**,**c**,**g**,**h**) bars = 10 mm; (**d**,**e**,**i**,**j**) bars = 2 mm; (**m**,**o**) bars = 40 µm; (**n**,**p**) bars = 20 µm; and (**k**,**l**) bars = 10 µm.

Tetraploid (2n = 4X = 36) plants were induced by colchicine (Figure 1l). Six tetraploid lines and six corresponding diploid plants were propagated by tissue culture after transfer to the experimental fields. A number of significant morphological changes were observed in each tetraploid plant (Figure 1 and Table 1). The leaf length and width of the tetraploid plants were significantly greater than for diploid plants, and the ratio of length to width was also greater. The leaves of all tetraploid plants exhibited a particular phenotype of thick, dark green, crimped and elliptic, distinct from the diploid plant leaves (Figure 1b,g). There was no significant difference in inflorescence diameter or disc flowers (all tubular florets) between diploid and tetraploid plants. The numbers of ligulate florets and tubular florets of tetraploid plants were reduced by 18.6% and 26.7%, whereas their dry weight increased by 40% and 54.5%, respectively, indicating that the ligulate and tubular florets of tetraploids were larger than for diploids. There was no significant difference in plant height between the diploid and tetraploid plants. Tetraploid plants leaves were thicker and larger than diploid plants. Increase in leaf thickness is due to increased palisade and spongy tissues thickness, increased by 9.3% and 9.9%, respectively (Table 1, Figure 1m,o). There is no difference between the upper and lower epidermis thickness. Number and size of palisade cell had significantly changed in third leaves of tetraploid and diploid plants. The mean cell size of palisade was increased 1.57 times, from 15.17 μm in the diploid plants to 23.86 μm in the tetraploid plants. The number of palisade cell was increased in tetraploid plants (from 138.99 × 10^4^ to 161.89 × 10^4^) (Table 1). Leaves become bigger resulting from the increase in number and size of cells.

### 2.2. Genomic Variation Induced by Autopolyploidy

**Figure 2:**
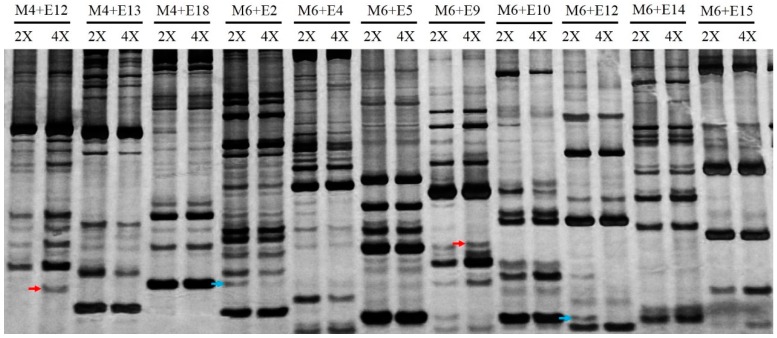
DNA-SRAP profiling of diploid and tetraploid of *C. lavandulifolium*: Upper panel represents primer combinations. 2X indicates diploid, and 4X indicates tetraploid. “→” to red arrows represents novel bands and blue arrows represent lost bands in the tetraploid. E# represents the em# primer, M# indicates me# primers. Bands detected range from 100 to 500 bp.

To investigate the genomic difference in diploid and tetraploid plants, SRAP was performed. Fragments present in the tetraploids but not in the diploids represented novel fragments, and fragments present in the diploids but not in the tetraploids represented lost fragments. Seventy-seven SRAP primer combinations produced a total of 440 bands. An average of 5.7 bands per primer pair were detected, with sizes ranging from 100 to 500 bp. Partial results are presented in Figure 2. Among these 440 loci, 1.6% (7/440) novel bands were found in the tetraploid *C. lavandulifolium,* whereas 1.1% (5/440) bands were absent compared with the diploid plants. These data indicate that there were genome changes from diploid to tetraploid.

### 2.3. Detection of Different Transcripts

**Figure 3:**
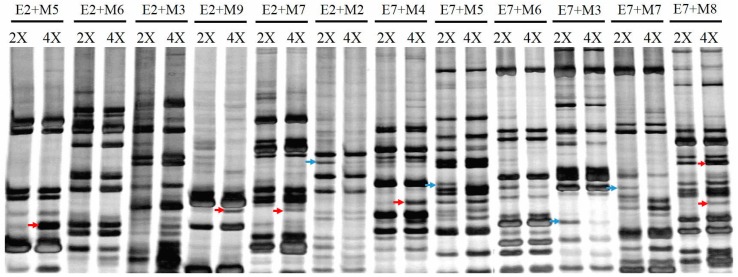
Typical cDNA-AFLP profiles of the diploid and the tetraploid. Upper panel represents primer combinations. 2X indicates diploid, and 4X indicates tetraploid plants. “→” to red arrows represent up-regulated genes and blue arrows represent down-regulated genes in tetraploid plants. Bands detected range from 100 to 500 bp.

To isolate differentially expressed transcripts, we performed cDNA-AFLP analysis on total RNA samples from the leaves of diploid and tetraploid *C. lavandulifolium.* Selective amplification with 56 primer combinations enabled visualization of 485 reproducibly detectable transcript-derived fragments (TDFs), with 8.7 TDFs detected per primer pair. Partial results are presented in Figure 3. Here, 62 fragments were detected as differentially expressed TDFs, corresponding to approximately 12.8% of all visualized transcripts. The differences in TDF intensity reflect fluctuations in the transcript levels. In tetraploid *C. lavandulifolium*, 33 differentially expressed TDFs were up-regulated, and 29 were down-regulated. One new fragment was produced with *EcoR* I-selective primer5 and *Mse* I-selective primer5 combinations in the tetraploids, which was absent in the diploids. 

### 2.4. Epigenetic Changes Induced by Autopolyploidy

**Figure 4:**
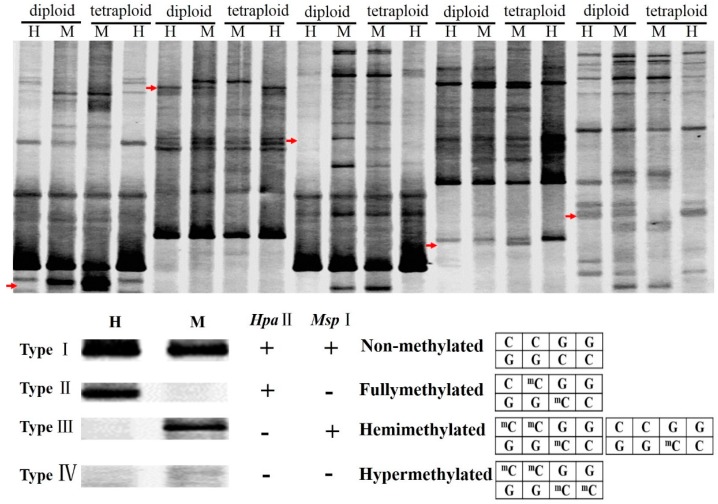
Representative variation in MSAP profiles. “→” to red arrows represents variation in DNA methylation between diploid and tetraploid plants; “+” represents fragments obtained after digestion with *EcoR* I or *Hpa* II/*Msp* I; “−” represents fragments not digested by *EcoR* I or *Hpa* II/*Msp* I; Type I fragments are nonmethylated and were presented in both the H (*EcoR* I or *Hpa* II digest) and M (*EcoR* I or *Msp* I digest) lanes; Type II are fully methylated and only appeared in the M lanes; Type III are hemimethylated and appeared in the H lanes; Type IV were fragments absent from both H and M lanes in diploid but present in either H or M lane of tetraploid, and vice versa.

Variations in methylation were explored using the MSAP technique. Based on the different methylation sites, methylation was divided into four types of fragments (Figure 4). Type I (non-methylated) fragments were shared by the H and M lanes, and Type II (fully methylated) fragments were only detected in M lanes, whereas Type III (hemi-methylated) fragments appeared only in the H lanes. The twenty-six primer combinations detected the DNA methylation levels of diploid and tetraploid *C. lavandulifolium*. In total, 61 fragments of Type I, 114 fragments of Type II, and 85 fragments of Type III were detected in the diploid plants, whereas 52, 109 and 90 such fragments were detected in the tetraploid plants, respectively (Table 2). The proportion of methylated fragments was 76.5% in diploid plants, which was slightly less than the proportion of 79.3% in tetraploid plants. This result suggested that the total levels of DNA methylation did not change during the process of autopolyploidization. A total of 53 fragments changed their methylation satatus between diploid and tetraploid *C. lavandulifolium*. Among them, 37 fragments exhibited increased methylation, and 16 fragments exhibited a decline in methylation (Table 3). Thus, approximately twice as many fragments exhibited increased methylation compared with decreased methylation. The satatus of increased methylation could be divided into two major types: shifts from Type III to Type IV and shifts from Type I to Type III. In addition, shifts from Type IV to Type III and from Type II to Type I were major forms of demethylation.

## 3. Discussion

### 3.1. Chromosome Number and Morphology Analysis

The leaf length and width of tetraploids significantly increased (Figure 1), but the inflorescence diameter had no significant differences between tetraploids and diploids. Compared with diploids, the dry weight of both florets (ligulate and tubular florets) was larger, but floret quantity was smaller in tetraploids. These phenomena were also observed in synthetic tetraploids of other species [30,31,32], the larger leaves of tetraploids was due to enlarged size and number of cells [33,34]. It indicates that the process of genome duplication was accompanied by enlarged cell size. The larger tubular and ligulate florets with few numbers of florets in tetraploids was likely attributable to the increased cell size of florets resulting from a decreased number of cells [35]. The increased leaf size and decreased number of florets from diploids to tetraploids in the present study suggest that individual organs became larger after polyploidization, rather than changes in the whole plant, which had been observed in maize [36]. Second division restitution (SDR) modes of 2*n* gamete were formed in process of tetraploid. A few fraction of epistasis were transmitted from the diploid parent to the teraploid plant with SDR to restrict to pairs of loci [37,38,39], and morphological changing of tetraploid plants may be part of reason.

### 3.2. Implications of Rapid Genome Change for Polyploidy Evolution

Autopolyploidy characterized by genomic redundancy and polysomic inheritance is extremely common in angiosperms [2,40]. Genomic composition and gene expression changed rapidly in both autopolyploids and allopolyploids. The changes in genome after polyploidy formation have received wide attention in allopolyploids, where the changes to the nuclear environment, such as gene silencing and activation, the loss and gain of parental restriction sites were more profound compared to the autopolyploids [1,25]. However, the limited evidence currently available suggests that autopolyploids experience strong genome recombination in early stages, and these processes may become more essential in the long run [24]. Here, we used SRAP to identify 440 fragments. Only seven were novel fragments (1.6%), and only five fragments (1.1%) disappeared in tetraploids. The genomic changes in the autopolyploid were far lower than in the allopolyploid [41]. These results were consistent with findings in the autopolyploids of weeping love grass and *Paspalum* [21,42,43]. At least two reasons might account for the loss or novel appearance of bands. First, these phenomena might result from retrotransposons that were transcriptionally activated in the process of polyploidization, leading to new genome modification [9,44]. Second, they might occur because of the elimination of DNA during genomic recombination [21], which could have altered the genomic structure to be more diploid-like to maintain the stability of the genome.

### 3.3. Induced Differences in the Transcriptome

Transcriptome studies of polyploidization in plants have revealed that the patterns of gene transcripts likely exerted a profound effect on the polyploidy context. In recent years, research in this area has focused largely on allopolyploid hybrids [45,46]. DNA methylation and demethylation, modifications of histones, transposon activation and insertion, small RNAs and RNAi and other factors may have been responsible for transcriptome differences in polyploids [9,16,47,48,49,50,51]. There have been fewer reports regarding autopolyploids [52]. Here, a total of 62 out of 485 (12.8%) complete transcripts were differentially expressed between diploid and tetraploid plants. Among these genes, 6.8% were up-regulated in expression, and 6.0% of genes were down-regulated in the leaves of tetraploid *C. lavandulifolium*. There were only a small number of changes in gene expression between ploidy levels, which was similar to previously reported results [25,53,54]. Many studies have demonstrated that differences in the transcriptome are associated with miRNA [55,56]. Many miRNA targets are transcription factors that were important to growth and development in plants. There were miRNA complete complimentary sequence to target gene, and target gene was degraded. miRNA loci may diverge with target gene and gain new expression patterns. miRNA regulation their targets gene may lead to novel phenotypes in the autotetraploids. In addition, we also found that a gene can be lost in the tetraploid plant, in agreement with some previous reports [57,58]. In our results, methylation occurred alongside up-regulation. Alterations in gene active or silence might be due to changes in the methylation or alteration of the DNA methylation patterns of a transcription factor/repressor, which could alter the expression of target genes without any further change of their methylation.

### 3.4 Methylation Alterations in Synthesized Autopolyploids

Methylation level can be rapidly altered after hybridization or allopolyploidization and it was inherited relatively stable. Methylation is essential for regulating plant development. In this study, the methylation levels were up to 76.5% and 79.3% in the diploid and tetraploid plants, respectively (Table 2). Although the genome doubled, the level of DNA methylation exhibited little change. The DNA methylation level is not closely associated with the ploidy level [59], and DNA methylation levels vary only slightly between tetraploid and diploid *Solanum* [60]. We suggest that the level of DNA methylation exhibits a weak relationship with the ploidy level in autopolyploids. Recent studies of autopolyploid plants have demonstrated that DNA methylation patterns may be associated with gene expression. It has been recognized that genes can be silenced by up-regulating methylation and turned on by down-regulating methylation in autopolyploids [22]. Here, the global pattern of methylation was adjusted between diploid and tetraploid plants, and the fragments with increased DNA methylation are 2.3 times as common as fragments with decreased methylation. Accordingly, it may be inferred that changes pattern of methylation caused by ploidy elevation may led to differential gene expression levels involved in the changed morphology of tetraploid plants. It should be noted that although a drop in DNA methylation might be induced by the callus phase [61]. The methylation changes in individual regenerants of *Codonopsis lanceolata* has been reported, but there was no significant global alteration in the DNA methylation during tissue culture [62]. There were some polymorphic bands in oil palm during tissue culture, but only 0.3% was recognized by the *Hpa* II [63]. The DNA methylation changes from tissue culture of rice were revealed using whole-genome bisulfite sequencing which only accounted for a small proportion of its genome [64]. In present study, the proportion of methylated fragments was 76.5% in diploid plants, which was slightly less than the proportion of 79.3% in tetraploid plants, which suggest that the changes in methylation level during autopolyploid is much higher than the proportion raised during the tissue culture. Therefore, we suggested that polyploidization may be the major driver of genomic and transcriptomic changes.

## 4. Materials and Method

### 4.1. Plant Material and Growth Conditions

The in vitro-cultured diploid *C. lavandulifolium* plantlets were cut into 2-cm-long young internodes, immersed in 200 mg/L colchicine, cultured for 36 h at a temperature of 25 ± 2 °C with shaking at 200 rpm. They were then rinsed three times in sterile water. Colchicine-treated plantlets were grown at 25 ± 2 °C under 16 h light/8 h dark provided by cool-white fluorescent lamps (30 µmol·m^−2^·s^−1^) on hormone-free Murashige and Skoog (MS) medium. After 30 days, the shoot internodes had developed lateral buds, we transferred them into rooting medium. Young root tips obtained from colchicine-treated plantlets were collected at 9:00–10:00 am, held in ice water for 20–24 h, fixed in ethanol:glacial acetic acid (3:1) (*v*/*v*) at 4 °C for 24 h, and then squashed in a drop of 45% acetic acid. The tetraploid and diploid were identified by observing mitotic chromosome spreads under optical microscopy. The both ploidy type plantlets were propagated and rooted in vitro and planted in a mixed medium of peat and perlite (3:1, *v*/*v*) and placed in the Chrysanthemum Germplasm Resource Preserving Centre, Nanjing Agricultural University, Nanjing, China.

### 4.2. Morphological and Anatomy Analysis

Six tetraploid lines and six corresponding diploid *C. lavandulifolium* were used for morphology analysis at 16 week old stage. Plant height, leaf length and width, leaf area (the third leaf below the apex), inflorescence, disc flower (flower central) diameter, dry weight of tubular florets and ligulate florets were determined. For anatomic observation, leaf segment (4 × 8 mm^2^) samples collected from middle part of third leaves were fixed in FAA (5% formaldehyde, 5% acetic acid and 63% ethanol (*v*/*v*/*v*) overnight at 4 °C). The samples were dehydrated with a graded ethanol series of ethanol solution and infiltrated with a xylene, then embedded in paraffin wax. Sections were cut to a thickness of 20–25 µm by a Leica RM2235 rotary microtome (Leica Instruments Company Ltd., Shanghai, China), and stained with toluidine blue. Then sections were observed and photographed under an Olympus BX41 microscope (Olympus Optical Company Ltd., Tokyo, Japan) and the leaf thickness, palisade thickness, spongy thickness, upper epidermis thickness and lower epidermis thickness from vertical sections were measured using imaging software (ScopeTek ScopePhoto3.0, Hangzhou, China). Palisade cells in the subepidermal layer of leaves were observed under a Nomarski differential interference contrast microscope (DMRXE; Leica Microsystems, Wetzlar, Germany) by following the experiment method of Horiguchi et al. [62]. The density of palisade cells per unit area of this region was determined, and the area of the leaf was multiply by this value to calculate the total number of palisade cells. For morphology, each line was repeated 9 times. In anatomy, three sections, which were cut from a leaf segment sample, were selected randomly, and then three fields of each section were measured. The data were presented as means± standard errors. SPSS v13.0 (SPSS, Inc., Chicago, IL, USA) and Microsoft Excel 2013 (Microsoft Corporation, Redmond, WA, USA) were used for statistical analysis (*p* < 0.05, Student’s *t*-test).

### 4.3. Genomic Variation Induced by Autopolyploidy

Total DNA was extracted from the leaves of three diploid and three tetraploid *C. lavandulifolium* lines using the Nuclei Isolation Kit (Solarbio, Beijing, China) according to the manufacturer’s instructions, respectively. An equal mixture of the DNA of the three lines constituted the gene pool for each ploidy level. The DNA pools were subjected to SRAP which was used to target open reading frames with 77 random combination of primers consisting of 14 forward primers (em) and 12 reverse primers (me) (Table S1). The SRAP-polymerase chain reaction (PCR) system (total of 20 μL) was as follows: 200 ng genomic DNA, 180 mM deoxynucleotide (dNTP), 0.5 mM of each primer, 2 mL 10× buffer (+Mg), and 2 U Taq polymerase (TaKaRa, Kyoto, Japan). The SRAP-PCR cycling parameters were as follows: 94 °C for 5 min; 5 cycles of 94 °C for 60 s, 35 °C for 60 s, and 72 °C for 120 s; 35 cycles of 94 °C for 60 s, 50 °C for 60 s, and 72 °C for120 s; and a final extension at 72 °C for 7 min, followed by preservation at 4 °C. The SRAP-PCR products were electrophoresed through 8% nondenaturing polyacrylamide gels, running at 240 V for 2.5 h in 1× TBE buffer, and were visualized via silver staining.

### 4.4. Complementary DNA Amplified Fragment Length Polymorphism Analysis

Total RNA was isolated from the third and fourth leaves of diploid and tetraploid *C. lavandulifolium* using the RNAiso reagent (TaKaRa, Osaka, Japan) with DNase treatment according to the manufacturer’s instructions. We constructed two separate RNA pools from diploid and tetraploid individuals. First-strand cDNA was synthesized from 1 μg of RNA using Moloney murine leukemia virus (M-MLV) reverse transcriptase (TaKaRa, Osaka, Japan) and an oligo-dT (18) primer. Double-stranded cDNA was synthesized using the Second Strand cDNA Synthesis Kit (Beyotime, Nanjing, China) according to the manufacturer’s instructions. Approximately 500 ng of double-stranded cDNA were subjected to standard AFLP template production according to Vos et al. [65]. The cDNA was digested with the restriction enzymes *EcoR* I and *Mse* I (New England Biolabs (NEB), Beijing, China) at 37 °C overnight. The digested products were ligated at 16 °C for 4 h. The ligation mixture contained the digested fragments, 10 pmol *EcoR* I and 100 pmol *Mse* I adapteors (Table S2), and 4 U T4 DNA polymerase (NEB). The enzymes were inactivated by heating to 65 °C for 20 min. Adapter-ligated DNA served as a template for pre-amplification, and the *EcoR* I and *Mse* I primers lacked any selective bases (Table S2). The diluted (30-fold) amplified products were used as the template for selective amplification, where the *EcoR* I and *Mse* I primers carried three selective bases (Table S2). The first selective amplification consisted of 12 cycles of 94 °C for 30 s, 65 °C for 30 s, and 72 °C for 60 s, in which the annealing temperature was lowered by 0.7 °C per cycle, followed by 23 cycles at 94 °C for 30 s, 56 °C for 30 s, and 72 °C for 60 s. The cDNA-AFLP products were electrophoresed in 8% nondenaturing polyacrylamide gels at 240 V for 2.5 h in 1× TBE buffer and then visualized via silver staining.

### 4.5. Mthylation Sensitive Amplified Polymorphism Analysis

The MSAP technique was applied to the DNA pools from three diploid lines and three tetraploid lines *C. lavandulifolium* They were digested with either *EcoR* I and *Hpa* II or *EcoR* I and *Msp* I (NEB) at 37 °C for 12 h. The digested fragments were ligated to 5 pmol *EcoR* I adaptor and 50 pmol *Hpa* II/*Msp* I adaptor by incubation with 4 U T4 DNA polymerase (NEB) at 16 °C for 4 has described for the AFLP method. Pre-amplification was performed using *EcoR* I and *Hpa* II/*Msp* I primers lacking any selective bases (Table S2), and selective amplifications were performed using *EcoR* I and *Hpa* II/*Msp* I primers carrying three selective bases (Table S2). The amplifications were electrophoresed through 10% denaturing polyacrylamide gels, running in 1× TBE buffer at 300 V for 3.5 h on ice, and then visualized by silver staining. We performed statistical analysis on the fragments between 100 and 500 bp. Every band was regarded as a genetic locus. The numbers “1” and “0” were used to indicate present and absent bands, respectively, to establish a database.

## Figures and Tables

**Table 1:** Morphology of diploid and tetraploid *Chrysanthemum lavandulifolium*.

Characters	Diploid	Tetraploid
Plant height (cm)	83.2 ± 2.2	85.13 ± 1.92
Leaf length (cm)	4.19 ± 0.20	4.73 ± 0.21 *
Leaf width (cm)	2.74 ± 0.13	3.50 ± 0.21 *
Inflorescence diameter (mm)	16.8 ± 0.24	16.65 ± 0.53
Disc flower diameter (mm)	7.73 ± 0.36	8.41 ± 0.25
No. of ligulate florets	13.11 ± 0.48 *	10.61 ± 0.86
Ligulate florets dry weight (mg/100)	0.018 ± 0.0015	0.03 ± 0.0012 **
No. of tubular florets	78.75 ± 1.97 **	57.71 ± 1.69
Tubular florets dry weight (mg/100)	0.015 ± 0.0014	0.033 ± 0.0010 **
Palisade thickness (µm)	43.39 ± 0.33	47.83 ± 1.85 *
Spongy tissue thickness (µm)	76.67 ± 1.01	85.16 ± 1.41 *
Upper epidermis thickness (µm)	8.51 ± 0.18	8.99 ± 0.32
Lower epidermis thickness (µm)	7.40 ± 0.17	6.86 ± 0.17
Leaf thickness (µm)	136.45 ± 1.16	148.84 ± 0.92 *
No. Cell (10^4^)	138.99 ± 22.49	161.89 ± 2.91 *
Size of cell	15.17 ± 0.36	23.86 ± 0.57 *

Data are presented as mean ± standard * *p* < 0.05 and ** *p* < 0.01 levels, based on *t*-test.

**Table 2:** Level of cytosine methylation in the autopolyploid.

Plant Lines	Total Sites	Nonmethylated Type I	Methylated		
			Type II	Type III	Total (II + III)
Diploid	260	61 (23.5%)	114 (43.8%)	85 (32.7%)	199 (76.5%)
Tetraploid	251	52 (20.7%)	109 (43.4%)	90 (35.9%)	199 (79.3%)

**Table 3:** Sataus of cytosine methylation in the autopolyploid.

Fragment Type		Fragment Display Pattern in MSAP Gel				Number of Sites	Sataus
2X	4X	2X H lane	2X M lane	4X H lane	4X M lane	2	↓
Type IV	Type I	−	−	+	+	0	↓
Type I	Type IV	+	+	−	−	2	↑
Type IV	Type III	−	−	+	−	7	↓
Type III	Type IV	+	−	−	−	12	↑
Type IV	Type II	−	−	−	+	4	↓
Type II	Type IV	−	+	−	−	5	↑
Type II	Type I	−	+	+	+	4	↓
Type I	Type II	+	+	−	+	5	↑
Type III	Type I	+	−	+	+	1	↓
Type I	Type III	+	+	+	−	13	↑

MSAP indicates methylation sensitive amplified polymorphism; “↓”decreased methylation, “↑” increased methylation.

## Supplementary Materials

Supplementary materials can be found at www.mdpi.com/1422-0067/17/10/1690/s1.
