# Supplementary material for: Morphological, Genome and Gene Expression Changes in Newly Induced Autopolyploid Chrysanthemum lavandulifolium (Fisch. ex Trautv.) Makino
Source: Int J Mol Sci. 2016 Oct 9;17(10):1690. doi: 10.3390/ijms17101690 (PMC5085722; doi:10.3390/ijms17101690)
Supplement: Supplementary file 1 [file ijms-17-01690-s001.pdf]

# Supplementary Materials: Morphological, Genome and Gene Expression Changes in Newly Induced Autopolyploid *Chrysanthemum lavandulifolium* (Fisch. ex Trautv.) Makino

**Ri Gao, Haibin Wang, Bin Dong, Xiaodong Yang, Sumei Chen, Jiafu Jiang, Zhaohe Zhang, Chen Liu, Nan Zhao and Fadi Chen**

## 1. Primer Combination of SRAP (Sequence-Related Amplified Polymorphism) (77)

em #4 plus me #4 (abbreviated"E4+M4")
M4+E4, M4+E6, M4+E11, M4+E12, M4+E13, M4+E18, M6+E2, M6+E4, M6+E5, M6+E9, M6+E10, M6+E12, M6+E14, M6+E15, M6+E17.
M7+E2, M7+E3, M7+E5, M7+E12, M7+E15, M7+E18, M8+E2, M8+E3, M8+E4, M8+E9, M8+E10, M8+E11, M8+E12, M8+E14, M8+E18.
M10+E2, M10+E3, M10+E4, M10+E5, M10+E9, M10+E10, M10+E11, M10+E12, M10+E17, M10+E18, M21+E2, M21+E3, M21+E4, M21+E5, M21+E10, M21+E11, M21+E12, M21+E14, M21+E15, M21+E17, M21+E18.
M22+E2, M22+E10, M22+E12, M22+E18, M23+E2, M23+E3, M23+E5, M23+E6, M23+E10, M23+E11, M23+E12, M23+E15, M23+E18, M25+E2, M25+E3, M25+E4, M25+E5, M25+E6, M25+E9, M25+E10, M25+E11, M25+E12, M25+E14, M25+E15, M25+E17, M25+E18.

**Table S1.** Sequences of primers for SRAP analysis.

| Primer | Sequence (5′–3′) | Primer | Sequence (5′–3′) |
|---|---|---|---|
| em2 | GACTGCGTACGAATTTGC | me4 | TGAGTCCAAACCGGACC |
| em3 | GACTGCGTACGAATTGAC | me6 | TGAGTCCAAACCGGTAG |
| em4 | GACTGCGTACGAATTTGA | me7 | TGAGTCCAAACCGGTTG |
| em5 | GACTGCGTACGAATTAAC | me8 | TGAGTCCAAACCGGTGT |
| em6 | GACTGCGTACGAATTGCA | me10 | TGAGTCCAAACCGGATG |
| em9 | GACTGCGTACGAATTACG | me21 | GTACATAGAACCGGAGT |
| em10 | GACTGCGTACGAATTTAG | me22 | TACGACGAATCCGGACT |
| em11 | GACTGCGTACGAATTTCG | me23 | CACAGTCATGCCGGAAT |
| em12 | GACTGCGTACGAATTGTC | me25 | CAGGACTAAACCGGATA |
| em14 | GACTGCGTACGAATTCAG | | |
| em15 | GACTGCGTACGAATTCTG | | |
| em16 | GACTGCGTACGAATTCGG | | |
| em17 | GACTGCGTACGAATTCCA | | |
| em18 | GACTGCGTACGAATTGAT | | |

"em#" and "me#" indicates name of forward primers and reverse primers.

## 2. Primer Combination of AFLP (Amplified Fragment Length Polymorphism) (56)

*Eco*RI selective primer #2 plus *Mse*I selective primer #5 (abbreviated"E2+M5")
E2+M5, E2+M6, E2+M3, E2+M9, E2+M7, E2+M2, E7+M4, E7+M5, E7+M6, E7+M3, E7+M7, E7+M8, E4+M9, E4+M6, E4+M5, E4+M4, E4+M2, E4+M7, E4+M8.
E9+M8, E3+M8, E3+M7, E3+M2, E3+M4, E3+M5, E3+M6, E3+M3, E3+M9, E5+M7, E5+M4 , E5+M5, E5+M6, E5+M3, E5+M9, E9+M7, E9+M2, E9+M4, E9+M5, E9+M6, E9+M3.
E8+M2, E8+M9, E8+M3, E8+M6, E8+M5, E8+M4, E8+M7, E6+M9, E6+M3, E6+M6, E6+M5, E6+M4, E6+M2, E6+M7, E6+M8, E8+M2.

## 3. Primer Combination of MSAP (Mthylation Sensitive Amplified Polymorphism)

*EcoR*I selective primer #9 plus *Hpa*II/*Msp*I selective primer #2 (abbreviated"E9+M2")
E9+H2, E9+H4, E9+H5, E9+H6, E9+H7, E9+H8, E9+H9
E6+H6, E6+H8, E6+H9, E8+H5, E8+H6, E8+H7, E8+H8, E8+H9,
E8+H2, E8+H3, E8+H4, E6+H2, E6+H3, E6+H4,
E4+H3, E4+H5, E4+H6, E4+H7, E4+H8

**Table S2.** Sequences of adaptors and primers used for pre-amplification and selective amplification in AFLP and MSAP analysis.

| Primer | Sequence (5′–3′) |
| --- | --- |
| *EcoR*I adaptor-F | CTCGTAGACTGCGTACC |
| *EcoR*I adaptor-R | AATTGGTACGCAGTCTAC |
| *Mse*I adaptor-F | GACGATGAGTCCTGAG |
| *Mse*I adaptor-R | TACTCAGGACTCAT |
| *Hpa*II/*Msp*I adaptor-F | GATCATGAGTCCTGCT |
| *Hpa*II/*Msp*I adaptor-R | CGAGCAGGACTCATGA |
| *EcoR*I pre-selective primer-1 | GACTGCGTACCAATTCA |
| *Mse*I pre-selective primer-1 | GATGAGTCCTGAGTAAC |
| *Hpa*II/*Msp*I pre-selective primer-1 | ATCATGAGTCCTGCTCGG |
| *EcoR*I selective primer-2 | GACTGCGTACCAATTCAAC |
| *EcoR*I selective primer- 3 | GACTGCGTACCAATTCAAG |
| *EcoR*I selective primer-4 | GACTGCGTACCAATTCACA |
| *EcoR*I selective primer-5 | GACTGCGTACCAATTCACT |
| *EcoR*I selective primer-6 | GACTGCGTACCAATTCACC |
| *EcoR*I selective primer-7 | GACTGCGTACCAATTCACG |
| *EcoR*I selective primer-8 | GACTGCGTACCAATTCAGC |
| *EcoR*I selective primer-9 | GACTGCGTACCAATTCAGG |
| *Mse*I selective primer-2 | GATGAGTCCTGAGTAACAA |
| *Mse*I selective primer-3 | GATGAGTCCTGAGTAACAC |
| *Mse*I selective primer-4 | GATGAGTCCTGAGTAACAG |
| *Mse*I selective primer-5 | GATGAGTCCTGAGTAACAT |
| *Mse*I selective primer-6 | GATGAGTCCTGAGTAACTA |
| *Mse*I selective primer-7 | GATGAGTCCTGAGTAACTC |
| *Mse*I selective primer-8 | GATGAGTCCTGAGTAACTG |
| *Mse*I selective primer-9 | GATGAGTCCTGAGTAACTT |
| *Hpa*II/*Msp*I selective primer-2 | ATCATGAGTCCTGCTCGGTAA |
| HpaII/MspI selective primer-3 | ATCATGAGTCCTGCTCGGTCC |
| *Hpa*II/*Msp*I selective primer-4 | ATCATGAGTCCTGCTCGGTTC |
| *Hpa*II/*Msp*I selective primer-5 | ATCATGAGTCCTGCTCGGTAC |
| *Hpa*II/*Msp*I selective primer-6 | ATCATGAGTCCTGCTCGGTGC |
| *Hpa*II/*Msp*I selective primer-7 | ATCATGAGTCCTGCTCGGTAG |
| *Hpa*II/*Msp*I selective primer-8 | ATCATGAGTCCTGCTCGGTTG |
| *Hpa*II/*Msp*I selective primer-9 | ATCATGAGTCCTGCTCGGTCA |

## 4. AFLP Marker

### *4.1. Protein AE7 (Daucus carota subsp. Sativus)*

GACTGCGTACCAATTCAACAATTCTATTCACCTGAGCTTATTGATCAGCTTGAGATATTTGA
ACATATTAGAGATATTAAGGATCCTGAGCATCCTTTTCTCTAGAAGATTTGAAAGTTATTAC
AAAGATGCTATTGAGGTAATGATAAGCGCGTTATGTGCGTGTTACTTTTACCCAACAGTTGA
GCACTCAGTATGGCAACGATTATTGGCTTTGTTTGCGGGTTAACTTATGCGTAGCCTTCCTTC

CCGTTTCAAGGTTGACATCAGGGTAGCGCCTGGGACTCATGCAACTGAAGATGCAGTGAAC
AAACAGTTGAACGATAATATGTTACTCAG

*4.2. Myb Domain Protein 73, Putative (Theobroma cacao)*

GACTGCGTACCAATTCACAAATTCACGACCCGCCCACCTCGTTGACTTTGTCGCTTCCTGGA
GCGGAGTCGAGTGAGGCTTCCTCGGCGGCTGTCACAGTGCCTGCGCCTCCGCCTGCCGTGG
CGGCGCCACCAGCTTTTGATGCGGAGTTTTTGGCGGTGATGCAAGAGATGATTAGAATGGA
AGTGAGGAATTATATTAGTGGTGATGGGTTTAGGGTTGGCGGCTGTGGGAAATAGGGAGAG
TCGTGGGTTTAGTTTATTTATTTTTTGTTTATGGTAAAACTAAATTAAATTTGTTGATGGTGAT
GATCAACATTTAGTTACTCAGGACTCATC

*4.3. No Apical Meristem (NAM) Protein (Cynara cardunculus var. scolymus)*

GATGAGTCCTGAGTAACTTTTCTCTATTTTCTTTGCTTTGAGGATACACAAATTATCCATTTT
GGGATGAGCATGAAACCAAACACAAACAACAGAAATGACATGGTGTAACGATGAAAACG
ATACCCTCCGAACTCTCTCAACTTCTTCAAGGAATCTCAAGAATAGTATCACAAGTTCATGT
CCTTCATGTGGACATGACATACAACTTCAAGCTAAGGGTTTGATTCATGATTTGCCGGGATT
ACCAGCTGGAGTGAAGTTCGACCCGACTGATCAAGAAATTCTTGAGCATTTGAAAGCCAAG
GTTGGATGTGATGCAGAAGAGCTTCATCCCCTTATCGATGAATTCATTCCAACTATTGAAGG
TGAAACTGGAATATGTTATACTCATCCTGAAAAATTACCTGGAGTTAGCAACGATGGCCAG
ATTCGCCATTTCTTTCACCGTCCATCCAAGGCTTACACCACAGGCACACGAAAGCGAAGAA
AGGTGCACAAATTCGACTGCGTACCAATTCAAC

*4.4. Transcription Factor, Teosint-Like1, Cycloidea and Proliferating Cell Factor( TCP ) (Cynara cardunculus var. scolymu)*

GACTGCGTACCAATTCACAAATTCGCCGCATCACTTTCATGGCGCTTTGGCGCTTGCTCCTC
ATCATCATCCTTTTGAAGAAGGGTTTTCTCAAATGATGGGCTTTCATCAGGGCCATGGTCAG
GGGCAGGTTCATGGTCAAGGGCAGCATATTATGACTGCTGACCAGATAGCTGAGGCGATTT
CGAGTGGTGGGGGTAATAATGGTGGAAATGGTGATGGGAGTGAGAATTATATGAGGAAAA
GGTATAGGGAGGATTTGTTTAAGGATGAGGGTGGTCAGAACAACGAGGTGGCGGTTGGAT
CACCGTCACCTTCGCCGTCTAACAAGGAGGGGAATACTGGTGCCGGAGCAGGGAATAGTA
TGCTCCGGCACCATGGGGGTATGATGCTTTGTTACTCAGGACTCATC

*4.5. Pol-Polyprotein (Silene Latifolia)*

TGAGTCCAAACCGGTGTTGGTAAAAAAGAGTGACGGAGGATGGCGCATGTGCGTGGATTTC
ACTGACATCAACAAAGCATGCCCTAAGGATTGCTACCCCCTACCCGAATTCGTACGCAGTC
